# Connecting Students’ Attitudes Toward Birds with Conservation Attitudes, Beliefs, and Knowledge Regarding the Grey Partridge (*Perdix perdix*)

**DOI:** 10.3390/ani14243665

**Published:** 2024-12-19

**Authors:** Ljubomir Mohorič, Ivan Kos, Vesna Mihelič Oražem, Iztok Tomažič

**Affiliations:** 1 Department of Biology, Biotechnical Faculty, University of Ljubljana, Večna pot 111, 1001 Ljubljana, Slovenia; ljubomohoric4@gmail.com (L.M.); ivan.kos@bf.uni-lj.si (I.K.); 2 Slovenia Forest Service, Večna pot 2, 1001 Ljubljana, Slovenia; vesna.orazem@zgs.si

**Keywords:** birds, grey partridge, students, attitudes, knowledge, reintroduction, conservation

## Abstract

As a farmland species, the grey partridge is facing a drastic decline all over Europe. In Vipava Valley (Slovenia), the species was last observed around 20 years ago. In this region, an initiative for reintroducing grey partridges was put forward, with much effort dedicated to breeding partridges and monitoring breeding success in a closed area. One of the initiative’s goals was to assess the local community’s attitudes, beliefs, and knowledge regarding birds and grey partridges. In this study, we investigated students’ attitudes toward birds in general, their attitudes toward and knowledge of grey partridges, and their beliefs about the feasibility of grey partridge reintroduction. Students generally supported the conservation of birds and grey partridges. However, they were neutral regarding learning about birds and undecided about the success of their reintroduction. Their beliefs regarding partridge reintroduction from the standpoint of natural heritage protection were slightly positive. The results showed that students were not familiar with the species. The study programme, willingness to volunteer, and amount of knowledge showed the most significant effects on students’ attitudes and beliefs. This study indicates that formal education should focus more on informing students about local biodiversity and related issues to raise local community awareness and engage them in conservation actions.

## Simple Summary

Many birds, especially farmland bird species, face drastic declines due to human actions. One of the possibilities for biodiversity conservation is the reintroduction of species where, besides ecological (biological) and legislative aspects, the social aspect should also be considered. Using a questionnaire, we assessed the attitudes and knowledge of general and vocational students (*N* = 576) regarding birds, in general, and grey partridges in particular. In general, students supported the conservation of all birds, but were not so eager to learn about them and were quite unsure about their reintroduction and its likelihood of success. Students’ knowledge about partridges was low. Study programme, willingness to volunteer (20% of all students), and amount of knowledge showed the most significant effects on students’ attitudes and beliefs. We highlight the importance of education about biodiversity and its conservation at the general and especially vocational (future stakeholders) school levels.

## 1. Introduction

The exceptionally rapid loss of biodiversity over the last few centuries indicates that a sixth mass extinction is already underway, driven primarily by anthropogenic factors [1]. According to the BirdLife International 2022 report, one in eight species of birds is threatened with extinction, and almost half of bird species worldwide have declining populations [2]. For the avifauna of the European Union, the overall breeding bird abundance is estimated to have declined by 17–19% since 1980, representing a loss of 560–620 million individual birds. The most significant declines in EU bird numbers are among species associated with agricultural land [3,4]. Similar trends have been recorded elsewhere [5,6].

On the IUCN Red List, the grey partridge (*Perdix perdix* Linnaeus, 1758) is categorised as a species of least concern, although most populations are in decline [7]. The grey partridge is a species found in agricultural ecosystems and was common in Slovenia in the last century [8]. After WWII, bird populations began to decline across Europe when land use changed agricultural ecosystems [7]. Several causes have contributed to their decline: the extensive use of pesticides, which can cause poisoning of partridges [9] or limit the availability of chick food supplies [7], as well as the increased roles of predation, hunting, early mowing, overwintering, and the time of first nesting [10,11]. In Vipava Valley, the last grey partridges (*P. perdix italica*) were reportedly observed between 2000 and 2005, almost 20 years ago. This subspecies of grey partridge is now presumed to be extinct in Slovenia [12].

To resolve this problem, corrective measures have been taken to restore the partridge populations, even in areas where the birds have almost become extinct [13]. Various studies and projects have been launched in many European countries to prevent the decline of natural partridge populations or increase their numbers [13,14,15,16]. Most of these studies have focused on environmental—specifically, habitat—changes that have historically contributed to the rapid deterioration and even collapse of entire populations. However, much less research has been devoted to the human aspects that we believe are the leading cause of the disappearance and extinction of the partridge from its European range. Kuijper et al. (2009) noted that, to restore partridge abundance, we “*should firstly focus on the main cause of population decline, that is, improve foraging conditions to increase chick survival rate. Next to the creation of special partridge habitat, conventional agriculture offers good opportunities to improve foraging conditions. Only when an integrative approach is adopted may large-scale habitat improvements be realised to restore population level to the level before 1950*” [7]. A human social aspect should also be considered to succeed in such endeavours; namely, the support and actions of local (farming) communities, conservation enthusiasts, and other interest groups can crucially contribute to the preservation of species [13].

One of the target groups that should be included in conservation actions is school students of different education levels and study programmes [17]. Students are present and future citizens who will, throughout their lives, impact the environment, or they are important future stakeholders in the conservation and management of species. To foster pro-environmental behaviours, it is of great importance to raise conservation-literate citizens [17,18]. While it is well documented that school activities that incorporate first-hand experiences with organisms and offer students active participation in learning can have a more positive effect on knowledge, skills, attitudes, and behaviours than traditional, teacher-centred approaches [19,20,21,22,23], they are often overlooked in the teaching of biology/science topics [24].

To date, research has shown poor knowledge and bird identification skills in different student groups [25,26,27] and in adults [28,29,30,31], which also applies to farmers regarding farmland bird species [32,33], where farmers from protected areas identified more birds than those from non-protected areas. Although most respondents in the research liked all bird species, bird identification ability correlated with the birds’ abundance and was influenced by participants’ gender and knowledge regarding birds’ ecological importance [32]. In the field of biology education, species identification skills have been proposed to be a prerequisite for learning about biodiversity [34,35]. Species knowledge can also influence environmental attitudes [36] and attitudes toward animals [37].

Research on attitudes toward animals has gained much attention in the last few decades. One widely used approach to assessing attitudes is Kellert’s attitudinal typology, which describes attitudes on ten dimensions (ecologistic, scientistic, utilitarian, negativistic, dominionistic, etc.) [38]. This attitudinal typology was widely used in the past for assessing attitudes toward different animals and animal groups [23,37,39,40,41] and for assessing attitudes toward birds [28,42], which was also used in the present study.

Research has shown that people generally have positive attitudes toward birds. If an individual is interested in birds, this can be linked to more knowledge about birds [31]. As for knowledge, numerous factors can influence attitudes toward species and their conservation, including gender [37,42,43], residency [37,44], and study level (study programme) [23,45], all of which were of interest in the present study. It was found in a Slovakian sample (aged 10–19 years) that knowledge of birds is positively related to attitudes toward birds. Younger students in that study had more knowledge than older ones [42].

Research on school students’ attitudes and knowledge about birds is, to the best of our knowledge, scarce and has mainly focused on bird identification skills [36], (mis)conceptions about birds [46], and the effectiveness of teaching on students’ knowledge about birds [47,48], with a few exceptions [25,42,49]. In one of the studies, the authors reported that almost 80% of children (79%) thought that female pheasants (*Phasianus colchicus*) were called partridges (*Perdix perdix*) [46]. Most studies regarding attitudes toward and knowledge of conservation and management of farmland birds, including grey partridges, are focused on adult rural populations (i.e., [32,50,51]), which is unsurprising, as farmland owners are directly engaged in agricultural practises.

On the contrary, students from different study levels and programmes will be future local or national decision-makers. Additionally, schools are an ideal place for informing students about various environmental issues, especially about the issues in local areas. In the last few years, an initiative for reintroducing grey partridges in Vipava Valley was launched, led by a dedicated individual who puts much effort into breeding partridges and monitoring their breeding success in a closed area. To gain insight into students’ attitudes toward birds in general, their attitudes toward and knowledge of grey partridges, and their beliefs about the feasibility of grey partridge reintroduction in Vipava Valley, we surveyed local primary, high, and vocational school students. To provide a theoretical background for the design of future communication and education activities related to the conservation of the species, we also included and evaluated several independent variables, namely, students’ gender, study level/study programme, place of residence (rural vs. urban), and reported willingness to volunteer in partridge reintroduction.

The following research questions (RQs) were explored in the present research:

1. **RQ 1:** Do sociodemographic factors (gender, place of residence, and reported willingness to volunteer in partridge reintroduction) influence students’ attitudes, beliefs, and knowledge regarding birds, particularly grey partridges?
2. **RQ 2:** Do students’ attitudes, beliefs, and knowledge regarding birds, particularly grey partridges, vary by study level and study programme (primary school, general high school, vocational preschool, and environmentalist and agricultural study programmes)?
3. **RQ 3:** Does the amount of knowledge influence the above-mentioned attitudes and beliefs?
4. **RQ 4:** Are there significant correlations between attitudinal dimensions and knowledge?

## 2. Materials and Methods

### 2.1. Study Design and Participant Information

Schools from the Vipava region and its surroundings were contacted, and permission to deliver the questionnaire was obtained from the schools’ head offices. In addition, biology teachers were asked if they would allow the researcher to administer the questionnaire to students within biology classes. Two primary schools declined participation, while one primary school and two high schools agreed to participate in the study. A convenience sample of 576 primary and high school students participated in the study (age: 11–20 years, *M* = 15.83 ± 1.61). The independent variables assessed were gender, place of residence, study level, and reported willingness to volunteer to reintroduce the species in Vipava Valley (Table 1). There were only a few cases of missing data within three independent variables. Participants were predominantly female (68.1%) and lived in a rural setting (61.1%). As the sample was unevenly distributed according to study level/study field, the results regarding this variable were interpreted with caution and should be further addressed in upcoming studies. In primary school, general high school, and vocational preschool education programmes, there were more females than males—55.6%, 73.4%, and 84.9%, respectively. Within the vocational environmentalist programme, there was almost an even number of males and females (47.1% females). In the agricultural programme, there were more males than females (72.4%). Such distribution is characteristic of the Slovenian school/programme population (https://cpi.si/en/, accessed on 2 December 2024). This study was conducted in a predominantly rural area, so the sample reflects the number of participants from rural areas.

The implementation of questionnaires took place in the 2022/2023 school year. At the beginning of each school year, schools in Slovenia gather consent from parents or caretakers for conducting surveys within school settings through an online system, eAsistent (“*I agree with the implementation of research activities and the distribution of survey sheets to students, i.e., student surveys, practitioners, …*”). As the survey was conducted for educational purposes, students’ personal data were not collected, and participation was voluntary. Ethical approval was not needed, according to Slovenian legislation.

### 2.2. Measures

For this study, a questionnaire that consisted of five parts was used (see Supplementary Materials—S2). In the first part, sociodemographic data were acquired. Next, students’ attitudes toward birds (interest in learning about birds and their attitudes toward bird conservation) were assessed using a 5-point Likert scale (BAQ: *N*_items_ = 8), followed by an assessment of their attitudes toward the importance of grey partridge (*Perdix perdix*) conservation and feasibility of its reintroduction into Vipava Valley because of altered habitats (farming, tourism, and predators). The same 5-point Likert scale assessed students’ attitudes (PAQ: *N*_items_ = 12). A colour photo of the animal was included in the questionnaire so that all students, regardless of their familiarity with the species, would rate their attitudes accordingly. The fourth part of the questionnaire assessed students’ beliefs regarding the reintroduction of grey partridges from the standpoint of natural heritage protection (PBQ: *N*_items_ = 9), again using a 5-point Likert scale. The last part of the questionnaire assessed students’ knowledge about grey partridge biology, ethology, and ecology and the reasons for local extinction (PKQ: *N* = 19). For this purpose, nine questions in the form of true/false statements and ten multiple-choice questions were used. For all questions, the option “*I do not know*” was used to minimise the possibility of guessing. For each statement or multiple-choice question, only one option was treated as correct. One point was assigned for the correct answer.

A panel of experts from the university, also experts in ornithology, checked the content validity of the questionnaire.

### 2.3. Data Analysis

After raw data were obtained, they were transferred to the SPSS 26.0 software (SPSS, IBM Germany, Ehningen), where all statistical procedures were conducted. For each scale, attitudes toward birds (BAQ), attitudes toward partridge conservation (PAQ), and beliefs regarding the reintroduction of grey partridge ratings (PBQ) were first submitted to principal component analysis (PCA) with direct oblimin rotation (Table 2, Table 3 and Table 4). Bartlett’s test of sphericity and the Kaiser–Meyer–Olkin (KMO) measure of the sampling adequacy test were calculated, with which the appropriateness of the datasets was assessed, with a critical KMO value of >0.70 and eigenvalues above 1.0 [52]. Principal component analysis (PCA) was applied to reduce the number of attitudinal/belief-dependent variables and to extract meaningful principal components (PCs). To test the reliability of the extracted PCs, Cronbach’s α coefficients were calculated. Cronbach’s α values below 0.69 show low internal consistency of the components and should be interpreted with caution [52].

Extracted PCs (from BAQ, PAQ, and PBQ) and summed knowledge scores (from PKQ) were used as dependent variables and further analysed according to independent variables.

Furthermore, average knowledge scores were used to categorise students into three groups: low (mean score < *M* − 1*SD*), middle (mean score within *M* ± 1*SD*), and high (mean score > *M* + 1*SD*) achievement (used as an independent variable). This categorisation compared the respondents’ attitudinal scores according to students’ achievements.

Descriptive (means and standard deviations) and inference (Mann–Whitney and Kruskal–Wallis tests) statistics were applied to find the source for emerging differences according to the independent variables. Bonferroni correction for multiple tests was used for multiple comparisons of the study level/field independent variable. Spearman’s correlation coefficients were calculated to assess the correlations between the dependent variables.

## 3. Results

In the first part, the results of the PCA of Likert-type scales are presented, followed by the effect of selected independent variables on students’ attitudes and knowledge. Additionally, knowledge was used as an independent variable when assessing its effect on attitudes. Spearman’s correlation coefficients between the dependent variables are presented in the third part of the results.

### 3.1. Results of the Principal Component Analysis (PCA)

To explore whether the use of PCA was appropriate for the Likert-type scales, the Kaiser–Meyer–Olkin (KMO) measure and Bartlett’s test of sphericity were calculated. The results of both tests showed that the PC structures of BAQ, PAQ, and PBQ were appropriate (see Table 2, Table 3 and Table 4). According to oblimin rotation, the Kaiser–Meyer–Olkin (KMO) measure of the sampling adequacy test (KMO_BAQ_ = 0.82, KMO_PAQ_ = 0.89, and KMO_PBQ_ = 0.92) and Bartlett’s test for sphericity (BAQ: *χ*^2^ = 976.11, *df* = 28, *p* < 0.001; PAQ: *χ*^2^ = 1636.28, *df* = 66, *p* < 0.001; PBQ: *χ*^2^ = 2044.87, *df* = 36, *p* < 0.001) suggested that the analysis was appropriate for these datasets.

Under BAQ, two meaningful principal components (PCs) were extracted: PC I—conservation of birds and PC II—interest in learning about birds (Table 2). PAQ also resulted in two meaningful PCs: PC I—conservation of partridges and PC II—reintroduction success (Table 3). The students’ beliefs regarding the reintroduction of grey partridges from the standpoint of natural heritage protection (PBQ) resulted in one principal component (Table 4). All except one calculated Cronbach’s α were satisfactory. Namely, PC II on PAQ showed a Cronbach’s α value of 0.57. Therefore, its results are discussed with caution.

Please see Appendix A for the descriptive statistics on BAQ, PAQ, PBQ, and PKQ.

### 3.2. Differences in Participants’ Attitude and Belief Ratings and Knowledge Score According to Independent Variables

#### 3.2.1. Differences in Participants’ Attitude and Belief Ratings and Knowledge Score According to Gender

According to gender, females displayed significantly more positive attitudes toward bird conservation than males, but males were slightly more positive regarding partridge conservation success (Table 5).

#### 3.2.2. Differences in Participants’ Attitude and Belief Ratings and Knowledge Score According to Place of Residence

Two small but statistically significant differences were also found regarding place of residence, where urban participants displayed slightly more positive attitudes regarding partridge conservation and the reintroduction of grey partridges from the standpoint of natural heritage protection beliefs (Table 6).

#### 3.2.3. Differences in Participants’ Attitude and Belief Ratings and Knowledge Score According to Study Level/Study Field

Education level/study field proved to have a greater significance on students’ ratings than gender and place of residence. Significant differences were found for all dimensions (Table 7), with the lowest differences on the dimension of “partridge reintroduction success”. A series of post hoc Mann–Whitney comparisons with Bonferroni corrections showed that primary school and vocational environmentalist study programme students had the most positive attitudes and were the most knowledgeable of all groups (Table 8). On the contrary, vocational preschool and agricultural programme students were the least knowledgeable and had more negative attitudes toward birds, in general, and toward partridges and their conservation. According to their ratings, general high school students were placed between these two groups.

#### 3.2.4. Differences in Participants’ Attitude and Belief Ratings and Knowledge Score According to Willingness to Volunteer in Species Reintroduction

The reported willingness to volunteer also proved to have a high significance on students’ ratings. Significant differences were found for all dimensions (Table 9), with the lowest differences on the dimension of “partridge reintroduction success”. In all cases, the participants willing to help with partridge conservation activities showed more positive attitudes toward birds and partridge conservation and were significantly more knowledgeable than their counterparts.

#### 3.2.5. Differences in Participants’ Attitude and Belief Ratings According to Knowledge Categories

Students’ level of knowledge also proved to have a significance on most students’ attitude/belief ratings (Table 10). The only exception was the students’ attitude toward partridge reintroduction success. Pairwise comparisons showed that students with low knowledge scores showed more negative attitudes and beliefs than students with medium and high knowledge scores. However, even medium achievers deferred from high achievers in interest in learning about birds and conservation attitudes regarding partridges.

### 3.3. Spearman’s Correlation Coefficients Between Dependent Variables

Spearman’s correlation coefficients between the dependent variables showed moderate-to-high correlations between all attitudinal and belief components except PAQ_PC II_, where the correlations were small (Table 11). Additionally, correlations between attitudinal/belief components and knowledge were smaller than those among attitudinal/belief components (except PAQ_PC II_). Students who were positive about bird conservation were also more interested in learning about birds and supported the conservation of partridges to a greater extent. They also rated the conservation of partridges higher from the standpoint of natural heritage protection. The highest correlation was found between the components of the “conservation of partridges—PAQ_PC I_” and the “conservation belief from the standpoint of natural heritage protection—PBQ”.

## 4. Discussion

This study compared students’ attitudes toward birds, attitudes toward grey partridge conservation and reintroduction, their beliefs regarding the reintroduction of grey partridges from the standpoint of natural heritage protection, and their knowledge of grey partridges.

### 4.1. General Findings

We found that the students generally supported the conservation of birds and grey partridges. However, they were, on average, neutral regarding learning about birds and undecided about the success of reintroduction. Their belief ratings regarding the reintroduction of grey partridges from the standpoint of natural heritage protection were, on average, slightly positive, and the average knowledge score of the total sample showed that the students were not familiar with the species. Although their attitudes supported the conservation of birds and partridges, their attitudes regarding the feasibility of reintroduction and their beliefs regarding reintroduction were unfavourable. The low knowledge of partridges can be linked to the 20-year absence of these birds from Vipava Valley, which corresponds with the students’ age. Namely, no student from our sample had seen a live partridge in a natural setting in their lifetime. Therefore, second-hand information about the species might have come from older people (i.e., parents or grandparents). Moreover, in Slovenian science and biology curricula, conservation biology topics are almost non-existent, and there is no learning goal dealing with the reintroduction of species. Therefore, the only source of information about species conservation might come from elsewhere (i.e., the media). One conservation example from recent years that also received high media coverage was rescuing the lynx population in Slovenia and Croatia from extinction by introducing additional, healthy animals from another population (https://www.lifelynx.eu, accessed on 2 December 2024).

Concerning independent variables, gender and place of residence had small effects on students’ attitudes, beliefs, and knowledge ratings.

### 4.2. The Effect of Gender

Females supported bird conservation more than males and were less convinced of the success of partridge reintroduction. However, the latter difference, although statistically significant, was very small. These results can be explained by females being more ecocentric than males [53] with higher pro-conservation attitudes toward birds. However, they supported the conservation of partridges as much as males. The lack of knowledge of the species in both genders might explain the latter. Additionally, awareness regarding one’s knowledge of the species could lead to lower beliefs about the success of reintroduction in females. Interestingly, there were no differences between males and females regarding interest in learning about birds, contrary to Hummel et al.’s study [54].

### 4.3. The Effect of Place of Residence

Place of residence (rural vs. urban) also produced small differences in the students’ ratings, where rural residents displayed less support for partridge conservation and did not believe in partridge reintroduction as much as urban residents. Here, it must be noted that there are no large cities or towns in Vipava Valley (less than 10,000 people per city). Therefore, a difference in students’ ratings and achievement was not evident. Randler and Heil noted that simply asking about rural vs. urban settings does not suffice, and it would be more important to ask about the distance to the next forest (or farmland, in the present case) [31].

### 4.4. The Effect of Willingness to Participate in the Reintroduction of the Species

On the contrary, the students’ willingness to participate in reintroduction actions and study level/programme produced significant differences in attitudes, beliefs, and achievement. Those students willing to participate in reintroduction actions were more positive in every attitude and belief dimension than their counterparts. Moreover, their knowledge was higher. Although only 20% of the participants expressed willingness to participate in the reintroduction of partridges, these results are encouraging because participants showed interest in the environmental issues and were more conservation-literate, all of which could lead to pro-environmental behaviour. There is a high chance that those students did not develop concern for the environment and knowledge in schools but from other sources. If schools would organise different locally relevant learning activities, such as participation in conservation actions, it could positively affect more students [20,48].

### 4.5. The Effect of Age and Study Programme

It turned out that younger students reported the most positive attitudes and beliefs regarding birds and partridges. The same applies to their knowledge. Research has shown that younger students express more positive attitudes because they are more willing to learn about animals [42] and are more sensitive regarding the conservation of animals [55]. The reason for higher achievement in primary school students can be found in the fact that, at this age, students learn about birds (though not directly about partridges) in schools. The same argument was proposed in other research [42,47]. Pairwise comparisons revealed that the most comparable groups were primary school students and vocational environmentalist programmes, followed by general high school, vocational agriculture, and vocational preschool study programmes. Interestingly, the primary school students displayed the highest amount of knowledge, probably because they were learning about birds in school at that age. The older students might have forgotten what they learned in primary school [42]. These results are similar to those gathered on attitudes and knowledge regarding wolves for Slovenian students [23,45]. Namely, students in different study programmes (i.e., veterinary technicians, environmentalists, and agricultural technicians) differ in attitudes and knowledge, where the most positive attitudes and the most knowledge were found in veterinary technicians, followed by environmentalists and agricultural technicians. The authors argued that the orientations and goals of individuals significantly affect their attitudes and knowledge of the species. The attitudes of agricultural technicians can be explained by their utilitarian views on animals and nature [38]. However, the low preschool education students’ attitudes and knowledge are somehow puzzling. Namely, these students will someday educate preschool children and have a great responsibility for raising awareness regarding environmental (biodiversity) issues. However, they seemed ambivalent regarding animal (nature) conservation and were not knowledgeable. The reasons for such results should be researched further. Furthermore, future studies should recruit a more uniform sample, according to the study programme, that would lead to more reliable conclusions.

### 4.6. The Effect of Knowledge on Students’ Attitudes and Beliefs

Level of knowledge also affected the students’ attitudes and beliefs ratings, except for attitudes toward reintroduction success. The students with low achievement scores rated the attitude and belief dimensions significantly more negatively than the students with medium and high knowledge scores. Although higher correlations were found between the attitudinal/belief dimensions, correlations between knowledge and the attitudinal/belief dimensions were still statistically significant. These results are similar to those regarding large carnivores [37,45], which shows some universality and points to the importance of education [19], where different vocational study programmes should not be neglected.

## 5. Conclusions

In the present study, general attitudes toward birds were linked with species-specific attitudes and knowledge in a school population, which is rare. This study provides insight into the importance of introducing locally relevant biodiversity issues to schools to raise students’ awareness and motivate them to take pro-environmental actions. This is especially true for vocational study programmes, vocational preschool, and vocational agricultural programmes. The first is important for educating the young, and the second for farmland management. It also indicates the importance of including such topics in school curricula (at a national level), where teachers can select topics that are also important locally. Evidently, the students in the present study were unfamiliar with grey partridges, and their attitudes were not species-based but, rather, based on a larger animal group—birds. Knowledge and willingness to volunteer in conservation actions and study programmes should not be neglected in future studies or when preparing educational activities on similar topics. However, the present study did not include variables such as media, community engagement, and parental influence, which could also be included in follow-up or other similar studies. Future studies should also assess the connections between attitudes toward and knowledge about species and environmental attitudes (values) through the application of already recognised questionnaires on the latter topic.

## Figures and Tables

**Table 1:** The distribution of participants (N_total_ = 576) according to independent variables (gender, place of residence, study level/field, and volunteering).

Independent Variable	Categories	*N*	*N* (%)
Gender	Male	182	31.6
	Female	392	68.1
	Not specified	2	0.3
Place of residence	Urban	220	38.2
	Rural	352	61.1
	Missing	4	0.7
Study level/field	Primary school	72	12.5
	General high school	244	42.4
	Vocational high school—preschool education	168	29.2
	Vocational high school—environmentalist	34	5.9
	Vocational high school—agriculture	58	10.1
Reported willingness to volunteer	Yes	124	21.7
	No	448	78.3
	Missing	4	0.7

**Table 2:** Principal component analysis (PCA) with an oblimin rotation of items on the bird attitude questionnaire (BAQ).

Item	Component (PC)	
	I	II
**Conservation**		
It is important to preserve birds in Slovenia for future generations.	0.800	
In our way of life, we should also consider the needs of birds.	0.777	
Birds should also have their own bird rights.	0.740	
Birds do not need to be preserved in Slovenia because most of these species also live elsewhere in Europe. (R)	0.412	
**Interest in learning**		
I love watching popular science shows about birds.		−0.928
I’d like to know the reasons why many species of birds are endangered.		−0.660
We should spend more time in schools learning about birds.		−0.625
I enjoy watching the birds in my surroundings.		−0.592
Kaiser–Meyer–Olkin (KMO)	0.821	
Bartlett’s test of sphericity	*χ*^2^ = 976.109 df = 28 *p* < 0.001	
Cronbach’s alpha	0.65	0.72
Eigenvalues	3.169	1.084
Explained variance (%)	39.61	13.55

Note: (R)—reversed items.

**Table 3:** Principal component analysis (PCA) with an oblimin rotation of items on the partridge attitude questionnaire (PAQ).

Item	Component (PC)	
	I	II
**Conservation**		
We must preserve the partridge population in the Vipava Valley for future generations.	0.785	
I want partridges to be present in the Vipava Valley, even though I may never observe or see them in nature.	0.746	
It is necessary to attract (include) nature lovers to conserve partridges.	0.744	
The reintroduction of partridges into the Vipava Valley could positively affect the natural balance of species.	0.711	
The reintroduction of partridges into the Vipava Valley is necessary but under appropriate professional guidance.	0.678	
As an alternative habitat, the suburban park could represent an important nature conservation, educational and tourist point for the entire Vipava Valley.	0.618	
As a society, we have a shared responsibility to conserve partridges.	0.602	
Partridges are so useful that this is reason enough for their reintroduction in the Vipava Valley.	0.557	
In the Vipava Valley, partridges do NOT need to be reintroduced because they already exist elsewhere in Slovenia and Europe. (R)	0.466	
**Reintroduction success**		
There are too many predators in the Vipava Valley (foxes, martens, buzzards, crows, goshawks, etc.) for the reintroduction of partridges to be successful. (R)		0.720
The landscape is too altered (agriculture, tourism, cycling, and recreation) for the reintroduction of partridges to succeed. (R)		0.683
The new conditions in agriculture (mass and night use of machinery) are unsuitable for reintroducing partridges. (R)		0.607
Kaiser–Meyer–Olkin (KMO)	0.893	
Bartlett’s test of sphericity	*χ*^2^ = 1636.281 df = 66 *p* < 0.001	
Cronbach’s alpha	0.84	0.57
Eigenvalues	4.140	1.474
Explained variance (%)	34.50	12.28

Note: (R)—reversed items.

**Table 4:** Principal component analysis (PCA) with an oblimin rotation of items on the partridge conservation beliefs questionnaire (PBQ).

Item	Component (PC)
	I
Pointless–sensible	0.799
Unacceptable–acceptable	0.759
Unimportant–important	0.750
Irresponsible–responsible	0.738
Unwise–wise	0.732
Useless–useful	0.715
Unprofessional–professional	0.688
Unnecessary–necessary	0.665
Uninteresting–interesting	0.632
Kaiser–Meyer–Olkin (KMO)	0.924
Bartlett’s test of sphericity	*χ*^2^ = 2044.872 df = 36 *p* < 0.001
Cronbach’s alpha	0.88
Eigenvalues	4.681
Explained variance (%)	52.010

**Table 5:** Effect of gender on students’ attitude/belief ratings and knowledge score.

Components	Males		Females		Mann–Whitney	
	*M*	*SD*	*M*	*SD*	*Z*	*p*
BAQ_PC I_	3.8	0.65	4.0	0.63	−3.304	0.001
BAQ_PC II_	2.9	0.79	2.9	0.78	−0.759	0.448
PAQ_PC I_	3.7	0.63	3.7	0.59	−0.470	0.638
PAQ_PC II_	3.1	0.64	3.0	0.56	−2.208	0.027
PBQ	3.6	0.69	3.6	0.62	−0.321	0.748
PKQ	5.7	3.40	6.0	3.18	−1.117	0.264

Note: BAQ_PC I_ = conservation; BAQ_PC II_ = interest in learning; PAQ_PC I_ = conservation of partridges; PAQ_PC II_ = reintroduction success; PBQ = reintroduction of grey partridges from the standpoint of natural heritage protection; PKQ = summed knowledge score.

**Table 6:** Effect of place of residence on students’ attitude/belief ratings and knowledge score.

Components	Urban		Rural		Mann–Whitney	
	*M*	*SD*	*M*	*SD*	*Z*	*p*
BAQ_PC I_	3.9	0.66	3.9	0.62	−0.741	0.459
BAQ_PC II_	2.9	0.84	2.9	0.75	−0.115	0.908
PAQ_PC I_	3.8	0.61	3.7	0.59	−2.119	0.034
PAQ_PC II_	3.1	0.59	3.0	0.58	−1.619	0.105
PBQ	3.7	0.68	3.6	0.61	−2.219	0.027
PKQ	5.8	3.28	6.0	3.22	−0.716	0.474

Note: BAQ_PC I_ = conservation; BAQ_PC II_ = interest in learning; PAQ_PC I_ = conservation of partridges; PAQ_PC II_ = reintroduction success; PBQ = reintroduction of grey partridges from the standpoint of natural heritage protection; PKQ = summed knowledge score.

**Table 7:** Effect of study level/field on students’ attitude/belief ratings and knowledge score.

Components	PS		GH		VP		VE		VA		Kruskal–Wallis		
	*M*	*SD*	*M*	*SD*	*M*	*SD*	*M*	*SD*	*M*	*SD*	*χ* ^2^	*df*	*p*
BAQ_PC I_	4.3	0.61	3.9	0.61	3.7	0.63	4.1	0.54	3.8	0.63	48.570	4	<0.001
BAQ_PC II_	3.2	0.94	2.9	0.78	2.7	0.74	3.0	0.68	2.9	0.67	20.096	4	<0.001
PAQ_PC I_	4.1	0.54	3.7	0.53	3.5	0.60	4.0	0.41	3.5	0.71	72.008	4	<0.001
PAQ_PC II_	3.0	0.63	3.1	0.59	2.9	0.53	3.0	0.55	3.1	0.67	12.116	4	0.017
PBQ	4.1	0.61	3.6	0.59	3.4	0.59	3.9	0.58	3.6	0.71	60.738	4	<0.001
PKQ	8.0	2.80	5.9	3.12	4.9	3.11	7.1	3.42	5.3	3.21	52.908	4	<0.001

Note: BAQ_PC I_ = conservation; BAQ_PC II_ = interest in learning; PAQ_PC I_ = conservation of partridges; PAQ_PC II_ = reintroduction success; PBQ = reintroduction of grey partridges from the standpoint of natural heritage protection; PKQ = summed knowledge score. PS—primary school; GH—general high school; VP—vocational preschool; VE—vocational environmentalist; VA—vocational agriculture.

**Table 8:** Effect of study level/field on students’ attitude/belief ratings and knowledge score: pairwise comparisons.

Components	Study Level/Field Pairwise Comparisons (Bonferroni-Corrected *p*-Values)									
	PS/GH	PS/VP	PS/VE	PS/VA	GH/VP	GH/VE	GH/VA	VP/VE	VP/VA	VE/VA
BAQ_PC I_	***	***		***	*			*		
BAQ_PC II_	*	***								
PAQ_PC I_	***	***		***	***	***				***
PAQ_PC II_					*					
PBQ	***	***		***				**		
PKQ	***	***		***	*			**		

Note: BAQ_PC I_ = conservation; BAQ_PC II_ = interest in learning; PAQ_PC I_ = conservation of partridges; PAQ_PC II_ = reintroduction success; PBQ = reintroduction of grey partridges from the standpoint of natural heritage protection; PKQ = summed knowledge score. PS—primary school; GH—general high school; VP—vocational preschool; VE—vocational environmentalist; VA—vocational agriculture. * *p* < 0.05; ** *p* < 0.01; *** *p* < 0.001.

**Table 9:** Effect of willingness to volunteer on students’ attitude/belief ratings and knowledge score.

Components	No		Yes		Mann–Whitney	
	*M*	*SD*	*M*	*SD*	*Z*	*p*
BAQ_PC I_	3.8	0.61	4.2	0.65	−6.403	<0.001
BAQ_PC II_	2.7	0.73	3.5	0.69	−9.467	<0.001
PAQ_PC I_	3.6	0.59	4.0	0.61	−5.791	<0.001
PAQ_PC II_	3.0	0.58	3.1	0.60	−2.282	0.022
PBQ	3.5	0.59	4.0	0.67	−7.117	<0.001
PKQ	5.7	3.29	6.7	2.90	−3.533	<0.001

Note: BAQ_PC I_ = conservation; BAQ_PC II_ = interest in learning; PAQ_PC I_ = conservation of partridges; PAQ_PC II_ = reintroduction success; PBQ = reintroduction of grey partridges from the standpoint of natural heritage protection; PKQ = summed knowledge score.

**Table 10:** Effect of achievement on students’ attitude/belief ratings.

Components	Low (L)		Medium (M)		High (H)		Kruskal–Wallis			Bonferroni-Corrected *p*-Values		
	*M*	*SD*	*M*	*SD*	*M*	*SD*	*χ* ^2^	*df*	*p*	L/M	L/H	M/H
BAQ_PC I_	3.6	0.68	3.9	0.61	4.1	0.62	29.205	2	<0.001	***	***	ns
BAQ_PC II_	2.6	0.64	2.9	0.78	3.2	0.82	31.082	2	<0.001	***	***	**
PAQ_PC I_	3.3	0.59	3.7	0.59	4.0	0.52	52.556	2	<0.001	***	***	**
PAQ_PC II_	3.1	0.43	3.0	0.61	3.0	0.60	1.781	2	0.410	ns	ns	ns
PBQ	3.2	0.59	3.6	0.62	3.8	0.65	38.807	2	<0.001	***	***	ns

Note: BAQ_PC I_ = conservation; BAQ_PC II_ = interest in learning; PAQ_PC I_ = conservation of partridges; PAQ_PC II_ = reintroduction success; PBQ = reintroduction of grey partridges from the standpoint of natural heritage protection; PKQ = summed knowledge score. ** *p* < 0.01; *** *p* < 0.001; ns—not significant.

**Table 11:** Spearman’s correlation coefficients between dependent variables.

Components	Spearman’s Rho				
	BAQ_PC II_	PAQ_PC I_	PAQ_PC II_	PBQ	PKQ
BAQ_PC I_	0.498 **	0.622 **	0.106 *	0.524 **	0.283 **
BAQ_PC II_		0.465 **	0.141 **	0.428 **	0.259 **
PAQ_PC I_			0.188 **	0.653 **	0.379 **
PAQ_PC II_				0.175 **	−0.045
PBQ					0.331 **

Note: BAQ_PC I_ = conservation; BAQ_PC II_ = interest in learning; PAQ_PC I_ = conservation of partridges; PAQ_PC II_ = reintroduction success; PBQ = reintroduction of grey partridges from the standpoint of natural heritage protection; PKQ = summed knowledge score. * *p* < 0.05; ** *p* < 0.01.

## Data Availability

The original contributions presented in this study are included in the Supplementary Materials. Further inquiries can be directed to the corresponding author(s).

## Supplementary Materials

The following supporting information can be downloaded at: https://www.mdpi.com/article/10.3390/ani14243665/s1, S1 (Dataset): The complete dataset for statistical analyses in the present study. S2 (Questionnaire): The questionnaire that was used in present research.

## Appendix A

**Table A1:** Descriptive statistics of individual attitude and belief items and summed knowledge score of the questionnaire.

Item	M	Me	SD	SE
**Birds—Conservation**				
It is important to preserve birds in Slovenia for future generations.	4.1	4.1	0.85	0.04
In our way of life, we should also consider the needs of birds.	3.6	3.6	0.87	0.04
Birds should also have their own bird rights.	3.7	3.7	1.06	0.04
Birds do not need to be preserved in Slovenia, as most of these species also live elsewhere in Europe. (R)	4.4	4.5	0.84	0.04
**Birds—Interest in Learning**				
I love watching popular science shows about birds.	2.3	2.3	1.10	0.05
I’d like to know the reasons why many species of birds are endangered.	3.2	3.2	0.98	0.04
We should spend more time in schools learning about birds.	2.6	2.6	1.07	0.04
I enjoy watching the birds in my surroundings.	3.5	3.5	1.10	0.05
**Partridge—Conservation**				
We must preserve the partridge population in the Vipava Valley for future generations.	3.9	4.0	0.89	0.04
I want partridges to be present in the Vipava Valley, even though I may never observe or see them in nature.	3.7	3.8	0.98	0.04
It is necessary to attract (include) nature lovers to conserve partridges.	4.0	4.0	0.90	0.04
The reintroduction of partridges into the Vipava Valley could positively affect the natural balance of species.	3.7	3.7	0.89	0.04
The reintroduction of partridges into the Vipava Valley is necessary but under appropriate professional guidance.	3.8	3.8	0.94	0.04
As an alternative habitat, the suburban park could represent an important nature conservation, educational and tourist point for the entire Vipava Valley.	3.5	3.5	0.93	0.04
As a society, we have a shared responsibility to conserve partridges.	3.6	3.6	0.98	0.04
Partridges are so useful that this is reason enough for their reintroduction in the Vipava Valley.	3.3	3.3	0.81	0.03
In the Vipava Valley, partridges do NOT need to be reintroduced because they already exist elsewhere in Slovenia and Europe. (R)	3.7	3.7	0.89	0.04
**Partridge—Reintroduction Success**				
There are too many predators in the Vipava Valley (foxes, martens, buzzards, crows, goshawks, etc.) for the reintroduction of partridges to be successful. (R)	3.0	3.1	0.87	0.04
The landscape is too altered (agriculture, tourism, cycling, recreation) for the reintroduction of partridges to succeed. (R)	3.2	3.3	0.87	0.04
The new conditions in agriculture (mass and night use of machinery) are unsuitable for reintroducing partridges. (R)	2.8	2.8	0.78	0.03
**Partridge—Conservation Beliefs**				
Pointless–sensible	3.6	3.6	0.91	0.04
Unacceptable–acceptable	3.8	3.9	0.92	0.04
Unimportant–important	3.6	3.6	0.90	0.04
Irresponsible–responsible	3.7	3.7	0.89	0.04
Unwise–wise	3.6	3.6	0.89	0.04
Useless–useful	3.7	3.7	0.85	0.04
Unprofessional–professional	3.6	3.6	0.88	0.04
Unnecessary–necessary	3.1	3.1	0.79	0.03
Uninteresting–interesting	3.6	3.6	0.99	0.04
**Knowledge**				
Summed score	5.9	5.9	3.24	0.14
