# Supplementary material for: Connecting Students’ Attitudes Toward Birds with Conservation Attitudes, Beliefs, and Knowledge Regarding the Grey Partridge (Perdix perdix)
Source: Animals (Basel). 2024 Dec 19;14(24):3665. doi: 10.3390/ani14243665 (PMC11727166; doi:10.3390/ani14243665)
Supplement: Supplementary file 1 [file animals-14-03665-s001.zip › S2-Questionnaire.pdf]

PARTRIDGE IN THE VIPAVA VALLEY

Purpose of the questionnaire
is to find out to what extent students are familiar with the problem of partridges in the Vipava Valley. With the survey, we want to find out what are your attitudes toward bird species, and especially partridges. We are particularly interested in the extent and how you are willing to participate in the project of reintroducing the partridge to the previously mentioned area. Please state your agreement or disagreement with the attitude statements and answer all questions about your knowledge of partridges.
We do not collect personal data of respondents with the questionnaire.

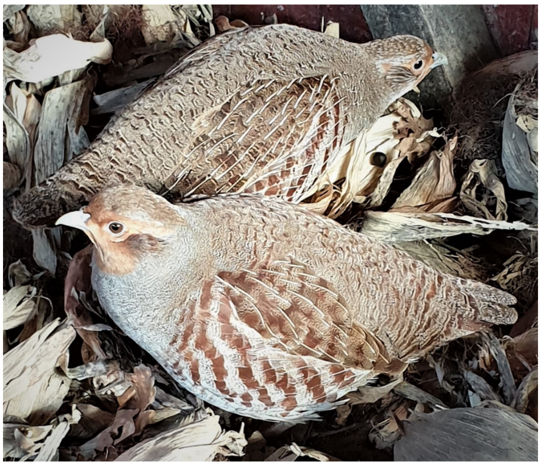

Photo: *Photo of an adult pair of partridges (Perdix perdix L.). Male in front, female in rear.*

For statistical data processing purposes, please fill in the fields below.

Class or year: 7 8 9 1 2 3 4 5

Gender: ☐ Female ☐ Male

Age: ___________ years.

Residence: [ ] urban, [ ] rural

**If I had the opportunity, I would volunteer to participate in partridge breeding and reintroduction.**	**YES NO**

I. State how much you agree with each statement about attitudes toward birds.

<u>**The Meaning of Values:**</u>

1 – Strongly Disagree, **2** – Disagree, **3** – Undecided, **4** – Agree, **5** – Strongly Agree

N	ATTITUDES TOWARD BIRDS	RESPONSE
1	Birds do not need to be preserved in Slovenia, as most of these species also live elsewhere in Europe.	1 2 3 4 5
2	I enjoy watching the birds in my surroundings.	1 2 3 4 5
3	I'd like to know the reasons why many species of birds are endangered.	1 2 3 4 5
4	In our way of life, we should also consider the needs of birds.	1 2 3 4 5
5	It is important to preserve birds in Slovenia for future generations.	1 2 3 4 5
6	Birds should also have their own bird rights.	1 2 3 4 5
7	I love watching popular science shows about birds.	1 2 3 4 5
8	We should spend more time in schools learning about birds.	1 2 3 4 5

Authors: Ljubomir Mohorič in Iztok Tomažič

II. If we wanted to reintroduce the partridge to the Vipava Valley, it would be from the standpoint of natural heritage protection in your opinion:

useless	1 2 3 4 5	useful
unwise	1 2 3 4 5	wise
unnecessary	1 2 3 4 5	necessary
irresponsible	1 2 3 4 5	responsible
unimportant	1 2 3 4 5	important
unacceptable	1 2 3 4 5	acceptable
uninteresting	1 2 3 4 5	interesting
pointless	1 2 3 4 5	sensible
unprofessional	1 2 3 4 5	professional

III. The table below lists the statements relating to your support for partridge conservation. Please rate your agreement with each statement on a scale of 1 to 5.

1 – Strongly Disagree, **2** – Disagree, **3** – Undecided, **4** – Agree, **5** – Strongly Agree

N	ATTITUDES TOWARD PARTRIDGES	RESPONSE
1	As a society, we have a shared responsibility to conserve partridges.	1 2 3 4 5
2	In the Vipava Valley, partridges do NOT need to be reintroduced because they already exist elsewhere in Slovenia and Europe.	1 2 3 4 5
3	I want partridges to be present in the Vipava Valley, even though I may never observe or see them in nature.	1 2 3 4 5
4	The reintroduction of partridges into the Vipava Valley could positively affect the natural balance of species.	1 2 3 4 5
5	We must preserve the partridge population in the Vipava Valley for future generations.	1 2 3 4 5
6	The new conditions in agriculture (mass and night use of machinery) are unsuitable for reintroducing partridges.	1 2 3 4 5
7	The landscape is too altered (agriculture, tourism, cycling, recreation) for the reintroduction of partridges to succeed.	1 2 3 4 5
8	There are too many predators in the Vipava Valley (foxes, martens, buzzards, crows, goshawks, etc.) for the reintroduction of partridges to be successful.	1 2 3 4 5
9	Partridges are so useful that this is reason enough for their reintroduction in the Vipava Valley.	1 2 3 4 5
10	It is necessary to attract (include) nature lovers to conserve partridges.	1 2 3 4 5
11	As an alternative habitat, the suburban park could represent an important nature conservation, educational and tourist point for the entire Vipava Valley.	1 2 3 4 5
12	The reintroduction of partridges into the Vipava Valley is necessary but under appropriate professional guidance.	1 2 3 4 5

IV. Knowledge of partridge issues. For each statement, define whether the statement is true or not true. If you don't know the answer, circle the don't know option.

N	STATEMENT	RESPONSE
1	A partridge is a useful bird for a farmer (livestock breeder, winegrower, fruit grower, farmer).	TRUE FALSE DON'T KNOW
2	The machining of agricultural land destroys many partridges.	TRUE FALSE DON'T KNOW
3	The partridge can disappear from the environment due to excessive disturbance (e.g. riding motorcycles, cycling, recreation - running, dog walking...) in its habitats.	TRUE FALSE DON'T KNOW
4	The partridge may disappear from the environment due to the construction of roads and bicycle paths.	TRUE FALSE DON'T KNOW
5	The partridge is a non-native species in Slovenia.	TRUE FALSE DON'T KNOW
6	Excessive hunting of the partridge can lead to its disappearance.	TRUE FALSE DON'T KNOW
7	A pheasant could replace a partridge on farmland.	TRUE FALSE DON'T KNOW
8	Many partridge juveniles die in cold and rainy springs.	TRUE FALSE DON'T KNOW
9	The partridge was exterminated in the past in the Vipava Valley.	TRUE FALSE DON'T KNOW

V. Please answer the questions that test your knowledge of partridges and related species (western capercaillie, black grouse, hazel grouse, rock partridge, and common quail). Circle only one answer for each question. If you don't know the answer, circle the "Not sure" option.

1) Are all of the above-mentioned forest and farmland species protected in Slovenia?
   a. Yes.
   b. No.
   c. Not sure.

2) Who in Slovenia endangers those species the most?
   a. Humans.
   b. Predators.
   c. Both.
   d. Not sure.

3) The species mentioned above are pests.
   a. Yes.
   b. No.
   c. Not sure.

4) How do partridges live?
   a. Solitary.
   b. In pairs.
   c. In flocks .
   d. In pairs or flocks – depending on the time of year.
   e. Not sure.

5) Partridges feed in nature:
   a. Mainly with food of animal origin (insects and their larvae).
   b. Mainly with food of plant origin (seeds and fruits).
   c. Food of animal and/or vegetable origin, depending on their age (chick, adult birds).
   d. Not sure.

6) Where does the partridge nest?
   a. On the trees.
   b. In the bushes (hedgerows).
   c. In the field among the grain.
   d. Not sure.

7) How many eggs does a partridge lay on average in nature?
   a. 10 – 15
   b. 15 – 20
   c. 20 – 25
   d. Not sure.

8) How long does a partridge hatch?
   a. 15 - 20 days.
   b. 24 - 25 days.
   c. 25 - 30 days.
   d. Not sure.

9) Which of the two parents takes care of the chicks after hatching?
   a. Female.
   b. Male.
   c. Both.
   d. Not sure.

10) Where does the partridge spend the night?
   a. On the tree.
   b. In the bushes (hedgerows).
   c. On the ground.
   d. Not sure.
